# Efficacy of Shenmai injection for the treatment of chronic heart failure: A protocol of systematic review

**DOI:** 10.1097/MD.0000000000020663

**Published:** 2020-06-26

**Authors:** Peng Gao, Xian Wu, Fu-hua Zhang, Zhi-li Qiao, Li-jie Yang

**Affiliations:** a Department of Emergency; b Department of General Medicine, Affiliated Hongqi Hospital of Mudanjiang Medical University, Mudanjiang, China.

**Keywords:** chronic heart failure, efficacy, safety, Shenmai injection

## Abstract

**Background:**

This study will assess the efficacy and safety of Shenmai injection (SMI) for the treatment of chronic heart failure (CHF).

**Methods:**

The following electronic bibliographic databases will be searched from inception to the March 25, 2020 without language and publication time limitations: MEDLINE, PUBMED, Cochrane Library, Web of Science, Scopus, WANGFANG, Chinese Biomedical Literature Database, and China National Knowledge Infrastructure. All randomized controlled trials related to the SMI for patients with CHF will be included. All study selection, data extraction, and study quality will be carried out by 2 reviewers. Any disagreements will be solved by a third reviewer through discussion. RevMan 5.3 software will be used for data synthesis and data analysis.

**Results:**

This study will summarize the present evidence of SMI for the treatment of patients with CHF.

**Conclusion:**

The findings of this study will determine whether SMI is effective and safety for the treatment of CHF or not.

**Study registration number:**

INPLASY202050029.

## 1 Introduction

Chronic heart failure (CHF) is a progressive clinical syndrome that results from insufficient blood supply from cardiac output to the metabolic need and accommodating venous return.^[1–3]^ Epidemiological studies showed that the prevalence of CHF is about 1% to 2% among general population,^[4,5]^ which results in high rate of mortality, hospitalization, poor quality of life, and poor prognosis.^[4,6,7]^ Thus, it is very important to prevent and treat patients with CHF.

Shenmai injection (SMI) has been widely utilized for the treatment of patients with CHF.^[8–12]^ Currently, there have been some clinical trials about the efficacy and safety of SMI therapy on CHF. However, most of these studies have low methodological quality ^[13–25]^ and no systematic review has conducted on this topic. Hence, this study aims to evaluate the efficacy and safety of SMI in patients with CHF systematically.

## 2 Methods

### 2.1 Study registration

This protocol has been registered at INPLASY202050029. This study will follow the guidelines of the Preferred Reporting Items for Systematic Reviews and Meta-Analysis Protocol statement.^[26,27]^

### 2.2 Inclusion criteria for study selection

#### 2.2.1 Types of study

This study will include randomized controlled trials (RCTs) in which SMI was used for the treatment of patients with CHF. Nonclinical studies and non-RCTs will be excluded.

#### 2.2.2 Types of participants

This study will include participants who were diagnosed as CHF irrespective sex, age, duration, and severity of CHF.

#### 2.2.3 Types of interventions

In the experimental group, all patients who underwent SMI will be included.

In the control group, there are no limitations to the comparators. However, we will exclude any forms of SMI as control interventions.

#### 2.2.4 Types of outcome measurements

Outcome measurements are all-cause mortality, urine output, serum sodium, and all expected and unexpected adverse events.

### 2.3 Strategy of literature retrievals

This study will retrieve the electronic bibliographic databases from inception to the March 25, 2020: MEDLINE, PUBMED, Cochrane Library, Web of Science, Scopus, WANGFANG, Chinese Biomedical Literature Database, and China National Knowledge Infrastructure. We will search above electronic databases without restrictions of language and publication time. We will consider all potential RCTs for inclusion that explored the efficacy and safety of SMI for patients with CHF. We will create a sample of search strategy for MEDLINE (Table 1). We will adapt similar search strategies for other electronic databases.

**Table 1:**
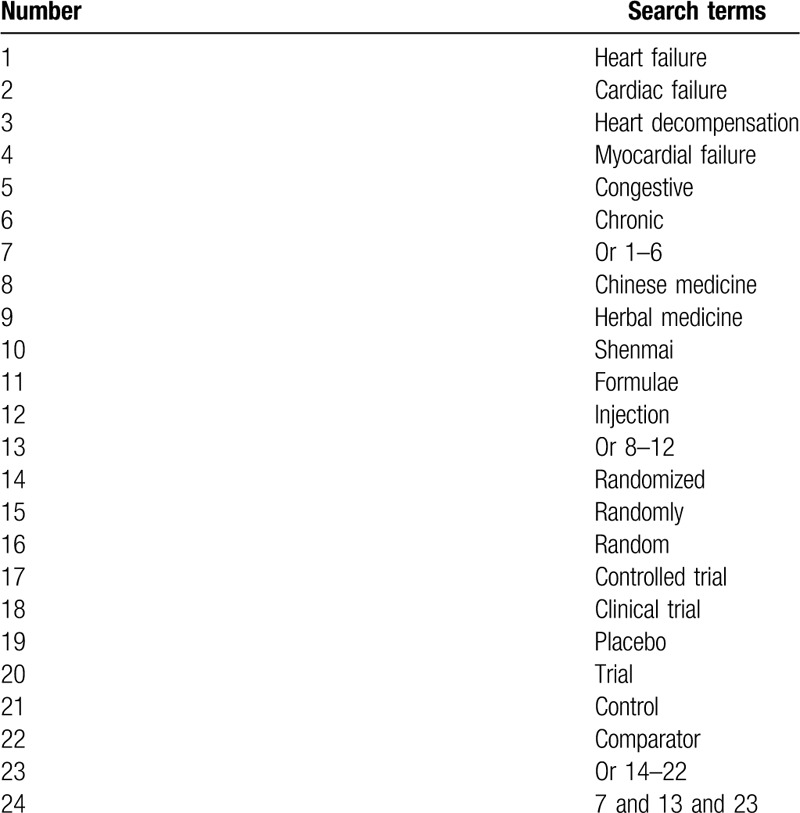
Search strategy of MEDLINE.

### 2.4 Data extraction and study quality assessment

#### 2.4.1 Study selection

Two independent reviewers will check titles/abstracts of all potential literatures, and will remove irrelevant studies. We will read full-text of potential trials to determine if they fulfill all inclusion criteria. Excluded studies will be listed with specific reasons in a table. Any divergences will be solved by consulting a third reviewer. The procedure of study selection is demonstrated in a flow chart.

#### 2.4.2 Data extraction and management

Two independent reviewers will extract essential data from all eligible trials using a predefined, pilot-tested extraction sheet. Any dissenting opinions will be resolved by a third reviewer. The essential information includes title, first author, country, time of publication, trial setting, trial design, diagnosed criteria, eligibility criteria, sample size, details of treatments and controls, outcome indicators, and adverse events. We will obtain any insufficient or missing data by email or fax from primary studies.

#### 2.4.3 Methodological study quality assessment

We will assess methodological study quality of each trial by 2 independent reviewers using Cochrane Risk of Bias Tool. If there are conflicts between both of them, we will invite a third reviewer to solve those issues through discussion.

#### 2.4.4 Measurements of treatment effect

In this study, we will estimate continuous data as weighted mean difference or standardized mean difference and 95% confidence intervals (CIs), and we will calculate dichotomous data as risk ratio and 95% CIs.

### 2.5 Statistical analysis

This study will use RevMan 5.3 software to pool and analyze extracted data. We will use *I*^*2*^ test to identify heterogeneity across trials. We defined it as follows: *I*^*2*^ ≤50% means acceptable heterogeneity, while *I*^*2*^ >50% suggests obvious heterogeneity. If *I*^*2*^ ≤50%, we will pool the data using a fixed-effects model. If minor heterogeneity is identified across trials, we will conduct a meta-analysis based on the sufficient similarity in study and patient characteristics, treatment and controls, and outcome indicators. If *I*^*2*^ >50%, we will pool the data using a random-effects model, and we will perform a subgroup analysis to examine the sources of obvious heterogeneity according to the different study characteristics, types of treatments and controls, and outcome measurements.

In addition, a sensitivity analysis will be performed to verify the stability and robustness of study findings by removing studies with low quality. We will examine the reporting bias by using funnel plot^[28]^ and Egg regression,^[29]^ if this study includes over 10 trials.

## 3 Discussion

CHF is a rising major cardiovascular disease globally. Although a variety of treatments are available for the treatment of CHF, the efficacy is still limited. SMI is reported to manage cardiovascular disease, especially for CHF. A large number of clinical trials reported that SMI can benefit patients with SMI. However, most of them have poor methodological quality, and their results are inconsistent. Therefore, it is very essential to critically assess the efficacy and safety of SMI for CHF. This study will systematically and comprehensively appraise the efficacy and safety of SMI for the treatment of patients with CHF. The results of this study may provide convinced evidence that can help to determine whether SMI is really effective and safe for the treatment of CHF.

### 3.1 Ethics and dissemination

This study will not require ethic approval, since no individual patient will be harvested. This study will be published on a peer-reviewed journal.

## Author contributions

**Conceptualization:** Peng Gao, Fu-hua Zhang, Zhi-li Qiao, Li-jie Yang.

**Data curation:** Xian Wu, Zhi-li Qiao, Li-jie Yang.

**Formal analysis:** Peng Gao, Zhi-li Qiao.

**Investigation:** Li-jie Yang.

**Methodology:** Peng Gao, Xian Wu, Fu-hua Zhang.

**Project administration:** Li-jie Yang.

**Resources:** Peng Gao, Xian Wu, Fu-hua Zhang, Zhi-li Qiao.

**Software:** Peng Gao, Xian Wu, Fu-hua Zhang.

**Supervision:** Li-jie Yang.

**Validation:** Peng Gao, Xian Wu, Fu-hua Zhang, Li-jie Yang.

**Visualization:** Peng Gao, Fu-hua Zhang, Li-jie Yang.

**Writing – original draft:** Peng Gao, Xian Wu, Zhi-li Qiao, Li-jie Yang.

**Writing – review & editing:** Peng Gao, Xian Wu, Fu-hua Zhang, Zhi-li Qiao, Li-jie Yang.
